# A School for the Study of Tropical Diseases

**Published:** 1898-07-16

**Authors:** 


### The Hospital

A Journal of

The Medical Sciences and Hospital Administration.

Vol. XXIV.—No. 616.] Edited by Sir Henry Burdett, K.C.B., and Solomon C. Smith, M.D., M.R.C.P. [July 16, 1898.

### A School for the Study of Tropical Diseases.

The proposal to establish in London a school for tropical diseases is not a matter which can be discussed entirely by itself. It is not an ephemeral and merely "happy" thought, but is the perfectly logical outcome of many social, scientific, and educational changes which have gradually been taking place during many years. First and foremost we have the growth of the imperialistic idea, the idea which we cannot but see is becoming a dominant one in regard to much that has to do with our external politics, namely, that this country and its colonies are linked together in a way our forefathers hardly thought of, and that while our foreign possessions furnish us with the sinews of commerce, we, on the other hand, are bound to furnish them with all that the arts and sciences can give to increase the comforts and amenities of life, and to lessen the dangers to health which have hitherto taken so heavy a toll from our colonists and explorers.

Another great change has arisen from the immense rapidity of scientific progress. This is visible on every side, and nowhere more so than in medicine, which within the active life of many of us has entirely altered its position among the sciences. From being an empirical art, in the practice of which experience was accepted as overriding all else, medicine has taken on the character of a science, in the learning of which grey-headed practitioners full of an over ripe experience willingly listen to the teachings of young laboratory professors.

Nothing can be more striking than the change which has come over the best practitioners of medicine in regard to willingness to learn—a change testified to by the large post-graduate classes which are to be met with in the great cities in Europe and America. The practitioner of old, if well educated to begin with, was thought only to lack experience —a lack that diminished with every year that went over his head; he did but want a fair start, which he got by "walking the hospitals," and his own observations were expected to be sufficient to serve him for his lifetime. How different all is now. Before men have been ten years in practice they complain that they are getting "rusty"; men in the army call out for "study leave;" men in the provinces regularly run up to town and "look in" at their old schools; and as for Americans, a whole regiment of them may be found, almost at any time, going round the main schools and hospitals of Europe—men who, after establishing practices and earning money, have thrown up all in order that they may see with their own eyes what there is to see, and may on their return start again on the new knowledge thus added to their store. On every hand, then, we see a recognition, both by the profession and the public, of the fact that education does not end at graduation, and that, every few years, knowledge must be renewed.

But there is another change, which, outside the profession, is perhaps hardly appreciated, and yet has a great bearing on the proposal for a School for Tropical Medicine, and is, indeed, the very parent of the idea, and that is the fact that it is impossible to teach the whole of medicine within the time allotted to the curriculum. Again and again within the lifetime of the older members of the profession has the time which must be passed at the medical schools been increased. But the new branches of study have increased more rapidly still, so that, hard as is the work, and difficult as are the examinations, it is admitted on all hands that there are whole fields of knowledge which are practically left out. Of these tropical medicine is one, and a most important one, for the lives of a great number of our countrymen and countrywomen, and those of a vast host of our fellow-subjects, depend on English medical men who practice in tropical regions possessing a good working knowledge of tropical diseases.

It is worthy of note that the study of tropical diseases touches all that is most recent in our knowledge of pathology. Much of the best of recent additions to medical science have had to do with parasitism, taking the term in a wide sense. But if there is one point in regard to tropical diseases more striking than another, it is the extent to which they are parasitic. Many of these ailments we never see in England, except at our great ports, where, down by the docks, sailors and Lascars and natives of many climes may be met with along with their parasites and the diseases caused by them. Dockwards, therefore, must we go if we would learn in England about tropical diseases and their effects. Nor must we forget that London has become the great port of England, the great mart of Western Europe, and that to London come samples of the inhabitants of every country within the tropics. When, then, Sir Henry Burdett, voicing the committee of the Dreadnought Seamen's Hospital Society, at Greenwich, wrote to the *Times* last Monday, describing and urging the claims of the School for the Study of Tropical Diseases which has been organised and is about to be opened in connection with their branch hospital situated between the Royal Victoria and Albert Docks, he did not give expression to a mere ephemeral ambition, or a mere temporary want, but described a movement which has originated in response to a necessity, a necessity which has arisen as a natural sequence from the progress of science and the progress of the empire. A School of Tropical Diseases London clearly ought to have, and that school should be at the Docks, where the results of those diseases and the sufferers from them are mostly to be met with.

